# Prevalence of Dementia among Malawian adults with HIV and without HIV: A Medical Record Review

**DOI:** 10.1002/alz.092252

**Published:** 2025-01-09

**Authors:** Haeok Lee, Yohannie Mlombe, Yeunjoo E. Song, Yun‐Beom Choi, Hyun‐Sik Yang, Joseph Maseke, Gyungah R Jun, Jonathan Ngoma

**Affiliations:** ^1^ New York University, Meyers College of Nursing, New York, NY USA; ^2^ Meyers College of Nursing, NY, NY USA; ^3^ Kamuzu University of Health Sciences, Blantyre, Southern Malawi; ^4^ Department of Population and Quantitative Health Sciences, Case Western Reserve University School of Medicine, Cleveland, OH USA; ^5^ Englewood Health, Englewood, NJ USA; ^6^ Rutgers New Jersey Medical School, East Orange, NJ USA; ^7^ Brigham and Women’s Hospital, Boston, MA USA; ^8^ Harvard Medical School, Boston, MA USA; ^9^ Daeyang University, Lilongwe, Central Malawi; ^10^ Boston University Chobanian & Avedisian School of Medicine, Boston, MA USA; ^11^ Kamuze Central Hospital, Lilongwe, Central Malawi; ^12^ Kamuze College of Medicine, Blantyre, Southern Malawi

## Abstract

**Background:**

Malawians, in Sub‐Saharan Africa (SSA), face the double burden of HIV and Alzheimer’s disease and related dementia (ADRD). The life expectancy among HIV‐positive people on antiretroviral therapy has increased consistently over the past 25 years. The purpose of the study was to determine the prevalence of dementia among people living with HIV (PLWHIV) and the general population without HIV as a comparison group in Malawi.

**Methods:**

We conducted a retrospective medical records review of four hundred consecutive patients from a single tertiary health center (200 PLWHIV from the HIV clinic and 200 without HIV from an outpatient clinic) in Lilongwe, Malawi. Since there was no electronic medical records system in Malawi, trained data collectors manually reviewed the medical records based on the instrument devices and the definitions between August 2023 and October 2023. We examined dementia prevalence and clinical characteristics including age, gender, and other clinical history between PLWHIV and the comparison group.

**Results:**

The PLWHIV group was significantly younger than the comparison group (mean age = 44.23±8.07 vs. 57.67±16.28; p<2e‐16), while there was no significant difference in sex (male 55% vs. 50%; p = 0.37). The overall prevalence of dementia was higher in PLWHIV than that in the comparison group (22% vs. 10%; p = 1.4e‐03 before adjusting for age and p = 1.9e‐07 after adjusting for age). Prevalence difference was higher in the older individuals (age>50; 39% vs. 13%; p = 3.2e‐4 before adjusting for age and p = 4.9e‐07 after adjusting for age). Dementia increased with age in both groups: 13% (age<45), 17% (45‐64), and 22% (65 and over), but more rapidly in PLWHIV than in the comparison group (17%, 28% and 50% vs. 5%, 4% and 20% respectively).

**Conclusion:**

This is the first study to demonstrate prevalence of dementia in PLWHIV was significantly higher than that of the general population in Malawi. We confirmed the increased risk of dementia in PLWHIV and provided valuable groundwork for future ADRD research in SSA, specifically in Malawi.

